# A Worksite Self-management Program for Workers with Chronic Health Conditions Improves Worker Engagement and Retention, but not Workplace Function

**DOI:** 10.1007/s10926-021-09983-6

**Published:** 2021-05-13

**Authors:** William S. Shaw, Robert K. McLellan, Elyssa Besen, Sara Namazi, Michael K. Nicholas, Alicia G. Dugan, Torill H. Tveito

**Affiliations:** 1 University of Connecticut School of Medicine, 263 Farmington Ave., Farmington, CT 06030 USA; 2 Geisel School of Medicine at Dartmouth, 1 Rope Ferry Drive, Hanover, NH 03755 USA; 3 Liberty Mutual Insurance, 175 Berkeley Street, Boston, MA 02116 USA; 4 University of Sydney at North Shore Hospital, St. Leonards, NSW 2065 Australia; 5 University of South-Eastern Norway, Raveien 215, 3184 Borre, Norway

**Keywords:** Self-management, Worksite intervention, Chronic health conditions, Work engagement, Work limitation

## Abstract

*Purpose* An increasing number of workers in the US have chronic health conditions that limit their ability to work, and few worksite interventions have been tested to improve worker coping and problem solving at work. The purpose of this study was to evaluate a worksite-based health self-management program designed to improve workplace function among workers with chronic health conditions. *Methods* We conducted a randomized, controlled trial of a worksite self-management program (“*Manage at Work”*) (clinicaltrials.gov #NCT01978392) for workers with chronic health conditions (N = 119; 82% female, ages 20–69). Most workers were recruited from the health care or light manufacturing industry sectors. Workers attended a 5-session, facilitated psychoeducational program using concepts of health self-management, self-efficacy, ergonomics, and communication. Changes on outcomes of work engagement, work limitation, job satisfaction, work fatigue, work self-efficacy, days absent, and turnover intention at 6-month follow-up were compared to wait-list controls. *Results* The most prevalent chronic health conditions were musculoskeletal pain, headaches, vision problems, gastrointestinal disorders, respiratory disorders, and mental health disorders. The self-management program showed greater improvement in work engagement and turnover intent at 6-month follow-up, but there was no evidence of a parallel reduction in perceived work limitation. Trends for improved outcomes of work self-efficacy, job satisfaction, and work fatigue in the intervention group did not reach statistical significance in a group x time interaction test. *Conclusions* Offering a worksite self-management program to workers with chronic health conditions may be a feasible and beneficial strategy to engage and retain skilled workers who are risking disability.

**Clinical trial registration**: Clinicaltrials.gov #NCT01978392.

## Introduction

An important and growing source of job stress for many workers is the challenge of managing one or more chronic medical conditions at work. Forty percent of US workers report persistent or recurrent musculoskeletal pain or other chronic physical health conditions that are currently limiting their ability to work [1, 2]. With the increased age and obesity of the US workforce, the prevalence of chronic diseases is likely to increase [3] and even younger workers are showing a higher prevalence of obesity and chronic health symptoms [4, 5]. The most prevalent chronic conditions impacting working-age adults include low back pain, arthritis, migraine headaches, depression, diabetes, heart disease, and asthma [6–8].

Chronic conditions can lead to unpredictable and fluctuating symptoms, daily oscillations in work tolerance and fatigue, increased injury risk, and complex self-care or treatment regimens [9–11]. For workers with chronic conditions, conventional health and disability employer benefits may fail to satisfy day-to-day needs for job flexibility, leeway, and organizational support to cope effectively with periodic symptoms and task limitations. Over time, this can lead to increased job stress, dissatisfaction, turnover, and long-term disability [12–18]. Persistent or intermittent symptoms and fluctuating dysfunction can challenge conventional workplace approaches to disability management and accommodation [11, 19–21].

While legislative mandates exist for employers to support job accommodation in principle, workers with chronic medical conditions still report problems communicating their needs effectively, making allowable work style and workstation adjustments, dealing with discomfort, overcoming stigma and discrimination, and keeping up with productivity expectations [22–24]. To overcome these problems, workers report a number of coping strategies: leveraging available job leeway and flexibility, careful planning and decision-making at work, obtaining sufficient job assistance and social support in and out of work, and communicating needs effectively and judiciously with peers and supervisors [25, 26]. In addition to its toll on workers, chronic illness increases employer costs through reduced productivity, high turnover rates, absenteeism, and health care expense [27–30]. To reduce these costs, more proactive strategies are needed for employers to help workers with episodic symptoms.

Besides objective work outcomes such as sickness absence and temporary disability leave, subjective self-report scales have been helpful to detect early perceptions of job-related limitations, disengagement, coping, and dissatisfaction among workers with chronic health conditions [15, 23, 29]. One hypothesis from this literature is that workers with chronic health conditions are unable to recover from a high daily level of exhaustion after working hours, and this ultimately leads to job burnout over time [23, 31]. Another theory is that workers slowly lose confidence in their abilities to perform routine tasks, to keep up with productivity demands, and to contribute meaningfully to their working teams [15, 30, 32]. These feelings may be associated with decreased personal self-efficacy and reduced confidence to manage pain or solve health-related challenges at work [32].

In the following study, we report the results of a randomized, controlled trial of a 5-session, worksite-based self-management program designed to address functional problems of workers with chronic health conditions (the *Manage at Work* study) [33]. We hypothesized that an employer-supported group intervention program adapted from principles of pain and illness self-management to the workplace context would improve work engagement and reduce work limitations.

## Method

### Participants

Participants were employees with chronic health conditions who expressed interest in a program designed to improve coping and function at work. Most were recruited from four worksites in the northeastern USA, including two large hospitals, a regional health care system, and a high-technology manufacturing firm. Participants were required to be full-time workers (> 20 h per week) and 18 years or older. Participants were required to have at least one chronic physical health condition (> 6 months) and most participants reported more than one chronic condition in a baseline survey (see "Results" sect. below). To avoid unnecessary health disclosure, participants were not required to provide medical information about diagnoses to qualify for the study. Reading and speaking in English was an inclusionary criterion. We excluded workers expecting to retire or change jobs within the next 12 months and workers who were unable to attend group workshops before work, after work, or during lunch breaks. Workers did not participate in the sessions during their standard, paid working hours.

### Procedure

Detailed study procedures, including steps of the intervention design process and a detailed list of survey measures, are described in a published study protocol [33] and in a clinical registry entry (clinicaltrials.gov, #NCT01978392). The study was publicized through posted flyers and email announcements sent to the entire workforce. Occupational health and safety staff also referred workers who described chronic health conditions at the time of a workplace injury or disability absence. Interested workers contacted the local on-site project coordinator who provided information about the study, answered questions, screened participants, obtained informed consent, and administered the baseline survey. The research office then provided randomization to the group intervention arm or to a wait-list control arm. A block randomization procedure was used (blocked in groups of 20) by the project statistician. After randomization, a local project coordinator contacted individuals in the treatment arm with a schedule for upcoming group intervention meetings. Control participants were placed on a waiting list for a one-day condensed workshop presentation of program materials one year later. Both control and intervention participants worked in the same occupational settings, so cross-contamination was a potential threat to internal validity, but information about study enrollment was kept confidential.

Research associates contacted participants in both the intervention and control groups 6 months after enrollment to complete a follow-up survey (20–30 min). Participants completed the baseline survey administered via an electronic computer tablet on-site in a private location. The 6-month follow-up surveys were completed on-line, with participants receiving an e-mail message prompt with an individual survey link and instructions on how to complete the survey. Participants in both the control and intervention groups received a $50 check for completion of each survey. The baseline survey asked participants for demographic information as well as baseline measures of covariates and main outcomes. The follow-up survey contained a similar battery of measures. There was no substantial deviation from the original study protocol, but funding for the project ended prematurely with the unexpected closing of the research institute sponsor. This resulted in a smaller than expected sample size and the inability to collect 12-month follow-up survey data.

### Group Intervention

The intervention program was developed using principles of health self-management programs but with messages tailored specifically to workplace challenges rather than lifestyle challenges at home. The goal was to improve personal perceptions of workability despite chronic health conditions and intermittent or episodic symptoms. The group workshop intervention content was developed based on qualitative studies [23] and review of existing self-management intervention elements [34]. The goal of the *Manage at Work* intervention was to provide coaching, education, and skill development to workers to help with self-management of chronic conditions in the workplace. The program was consistent with existing pain and illness self-management programs; however, the key messages and discussions were focused on overcoming workplace functional challenges. Workshop topics included coping, modifying work, communicating effectively, dealing with negative thoughts and emotions, and self-management of overall health and well-being. The sessions included presentations by the facilitator, group discussions, self-assessment activities, and brief homework assignments.

Five specially trained group facilitators led group management workshops. Group facilitators were licensed psychologists or clinical social workers, some of whom were providing services as part of the company’s Employee Assistance Program. Group meetings were apportioned differently, with some groups opting for five 2-h sessions and others preferring ten 1-h sessions or seven 1.5-h sessions, depending on the schedules and availability of participants. Group sizes varied from 3 to 10. EAP professionals participating as group facilitators reported productive group discussions and high participation levels of study participants, and facilitators voiced no major feedback or concerns about program content and format to the research investigators.

### Measures

#### Primary Outcome Measures

Two workplace-relevant self-report scales were chosen as primary outcome measures to reflect two different perspectives on workplace function. The Work Limitations Questionnaire (WLQ) [35] is a 25-item self-report questionnaire that assesses the degree to which working individuals are experiencing limitations on-the-job due to their health problems and health-related productivity loss. Respondents rate the level of difficulty or ability to perform specific job demands including time management, physical demands, mental-interpersonal demands, and output demands. The WLQ responses are on a 5-point scale from “1” (all of the time) to “5” (none of the time). The scale has good internal consistency [36] and has been validated against other health and disability constructs. Scores can be translated into a single Productivity Index score that estimates the percentage loss in work output due to health concerns. The Utrecht Work Engagement Scale (UWES) was chosen as a second primary outcome measure to provide an alternative perspective on functionality at work. The short-form of the UWES [37] is a 9-item self-report questionnaire designed to measure the degree to which employees have a sense of energetic connection with their work activities and see themselves as able to deal with the demands of their job. The UWES responses are on a 7-point scale from “0” (never) to “6” (always or every day). The UWES has good psychometric properties [38] and captures more holistic views of work related to vigor, dedication, and absorption.

#### Secondary Outcome Measures and Covariates

To provide a more in-depth collection of work outcomes in this population, we added other self-report scales encompassing workplace fatigue (the Occupational Fatigue Exhaustion Recovery [OFER] scale [39, 40]), turnover intention [41], job satisfaction [42], a work self-efficacy measure combining items from the Pain Self-Efficacy Questionnaire [43] and Return-to-Work Self-Efficacy [RTWSE-19] scale [44]. Details of these scales and their scoring and psychometric properties are included in the published study protocol [33].

### Data Analyses

As a first step in the analyses, baseline demographic and health variables were compared between the intervention and control groups to test whether randomization had produced two equivalent groups. Any variables that showed statistically significant group differences were retained as covariates in subsequent analyses. The primary analytic strategy was to compare the intervention and control groups on changes in outcome measures (WLQ and UWES) at 6-month follow-up using a repeated measures general linear model, with group assignment being the between-subjects factor and time (baseline vs. follow-up) being the within-subjects factor and including any necessary demographic covariates. In the repeated measures model, a statistically significant group x time interaction term (p < 0.05) was evidence of different group outcomes. All analyses were conducted using IBM SPSS Statistics for Windows [45].

## Results

A total of 119 participants (98 female, 20 male, 1 unspecified gender) provided informed consent and were randomized to the intervention or control arms of the study. The most common occupations were administrative assistant (19%), manager/supervisor (17%), data analyst or research assistant (13%), medical assistant (12%), medical technologist (9%), nurse or nursing assistant (7%), and coding or billing specialist (7%). Other occupations included lab scientists, engineers, teachers, cashiers, assemblers, and counselors. The study population was comprised of mostly skilled workers in technical jobs with at least moderate levels of job security and opportunities for advancement.

Participants endorsed a median of 3 chronic health conditions (range 1–11). The most frequent chronic conditions were as follows: back or neck problems (85%), hand/arm problems (61%), leg or feet problems (55%), migraine or severe headaches (43%), vision problems (31%), stomach or bowel disorders (24%), asthma, bronchitis, or emphysema (22%), mental health disorders (17%), hearing problems (12%), cardiovascular disease (5%), severe skin disorders (4%), and diabetes (4%). Participants responded to either employer-based (92%) or community-based (8%) announcements about the study. The most prevalent comorbidity was to report a chronic musculoskeletal pain problem affecting more than one body part. Most participants (83.2%) were working in a healthcare setting. Participants reported missing from 0 to 70 workdays (M = 5.7, SD = 12.1) due to chronic health conditions over the previous six months.

Table 1 shows demographic characteristics by study groups, and these data reflect a mostly White, college educated, middle-aged cohort of workers with significant industry experience and job tenure. Despite random assignment to the intervention and control arms of the study, there was a trend for the control group to be older (mean age = 47.9 versus 44.2) and there was a statistically significant group difference on job tenure, with the control group having more workers with > 5 years (61% versus 42%). Because these two variables have implications for both work and health outcome variables, they were retained as covariates in the subsequent multivariate group comparisons of study outcomes. There were no statistically significant differences or notable trends between the control and intervention groups on any other demographic variables.

**Table 1:** Baseline demographic characteristics of study participants by randomized group assignment

	All participants (n = 119)	Intervention group (n = 60)	Control group (n = 59)	*t* or *χ* ^2^	*p*
	M (SD)/n (%)	M (SD)/n (%)	M (SD)/n (%)		
Age	46.0 (12.7)	44.2 (12.0)	47.9 (13.2)	*t* = 1.624	0.107
Chronic health conditions	3.7 (1.9)	3.7 (2.1)	3.6 (1.7)	*t* = 0.208	0.836
Children/dependents at home				*χ*^2^ = 1.66	0.799
Yes	45 (37.8)	22 (36.7)	23 (39.0)		
No	74 (62.2)	38 (63.3)	38 (64.4)		
Gender				*χ*^2^ = 3.16	0.206
Male	20 (16.8)	13 (21.7)	7 (11.9)		
Female	98 (82.4)	46 (76.7)	52 (88.1)		
Not specified	1 (0.8)	1 (1.7)	0 (0.0)		
Race				*χ*^2^ = 0.17	0.918
Asian	2 (1.7)	1 (1.7)	1 (1.7)		
Black	7 (5.9)	3 (5.0)	4 (6.8)		
White	108 (90.8)	55 (91.7)	53 (89.8)		
Not reported	2 (1.7)	1 (1.7)	1 (1.7)		
Ethnicity				*χ*^2^ = 0.00	1.000
Hispanic	4 (3.4)	2 (3.3)	2 (3.3)		
Non-Hispanic	112 (94.1)	56 (93.3)	56 (94.9)		
Not reported	3 (2.5)	2 (3.3)	1 (1.7)		
Marital status				*χ*^2^ = 2.21	0.531
Never married	25 (21.0)	12 (20.0)	13 (22.0)		
Married/partnered	67 (56.3)	35 (58.3)	32 (54.2)		
Divorced/separated	25 (21.0)	13 (21.7)	12 (20.3)		
Widowed	2 (1.7)	0 (0.0)	2 (3.4)		
Annual income				*χ*^2^ = 5.00	0.840
$10,000–$19,999	3	1	2		
$20,000–$29,999	14	8	6		
$30,000–$39,999	27	12	15		
$40,000–$49,999	27	15	12		
$50,000–$59,999	11	7	4		
$60,000–$69,999	10	5	5		
$70,000–$79,999	13	5	8		
$80,000–$89,999	5	2	3		
$90,000–$99,999	4	1	3		
$100,000 or over	4	3	1		
(Missing)	1	1	0		
Highest education				*χ*^2^ = 3.69	0.450
< 12 years	1 (0.8)	0 (0.0)	1 (1.7)		
High school	9 (7.6)	6 (10.0)	3 (5.1)		
Some college	54 (45.4)	28 (46.7)	26 (44.1)		
Bachelor’s degree	27 (22.7)	15 (25.0)	12 (20.3)		
Post-bachelor’s	28 (23.5)	11 (18.3)	17 (28.8)		
With current employer				*χ*^2^ = **14.17**	**0.015**
0–6 months	8 (6.7)	4 (6.7)	4 (6.8)		
6–12 months	8 (6.7)	8 (13.3)	0 (0.0)		
1–2 years	8 (6.7)	6 (10.0)	2 (3.4)		
2–5 years	34 (28.6)	17 (28.3)	17 (28.8)		
5–10 years	29 (24.4)	9 (15.0)	20 (33.9)		
> 10 years	32 (26.9)	16 (26.7)	16 (27.1)		
With current industry				*χ*^2^ = 3.67	0.599
0–6 months	2 (1.7)	0 (0.0)	2 (3.4)		
6–12 months	5 (4.2)	4 (6.7)	1 (1.7)		
1–2 years	10 (8.4)	5 (8.3)	5 (8.5)		
2–5 years	24 (20.2)	14 (23.3)	10 (16.9)		
5–10 years	26 (21.8)	12 (20.0)	14 (23.7)		
> 10 years	52 (43.7)	25 (41.7)	27 (45.8)		
Working > 1 employer				*χ*^2^ = 0.01	0.973
No	105 (88.2)	53 (88.3)	52 (88.1)		
Yes	14 (11.8)	7 (11.7)	7 (11.9)		

Attendance records maintained by group facilitators reflected a moderate to high level of engagement in the intervention group, with 81% of participants completing at least half of the workplace self-management sessions. Subjective ratings of satisfaction with the intervention program were also high (mean overall satisfaction rating of 8.8 out of 10). Participants reported no adverse events related to study participation.

The means and standard deviations for baseline and follow-up measures are shown in Table 2. Of the original sample (n = 119), 98 participants (82%) completed the 6-month survey. All tests of statistically significant changes in groups are controlled for the baseline covariates of age and job tenure. Both groups showed increasing work limitations over the 6-month period, and there was no significant group x time interaction (p > 0.05) that would suggest the intervention program had attenuated this effect. On work engagement, however, the intervention group improved slightly, while the control group experienced a decline over the same 6-month period (interaction test, *p* < 0.05). There was also a statistically significant improvement in turnover intent for the intervention group versus the control group (interaction test, *p* < 0.05). Secondary measures of work self-efficacy, work fatigue, and job satisfaction showed a trend for the intervention group to improve in contrast with the control group, but these differences did not reach statistical significance.

**Table 2:** Changes in work outcomes by group assignment

Variable	Intervention group (*n* = 47)^a^			Control group (*n* = 51)^b^			Group x time interaction^c^	
	Baseline M (SD)	6-month M (SD)	Effect size (Cohen’s d)	Baseline M (SD)	6-month M (SD)	Effect size (Cohen’s d)	*F*	*p*
Primary outcome measures								
Work engagement	4.53 (0.99)	4.79 (1.36)	+ 0.22	5.10 (1.01)	4.68 (1.32)	− 0.36	**7.88**	**0.006**
Work limitation	7.66 (4.80)	8.15 (5.16)	+ 0.10	6.79 (4.90)	7.77 (5.51)	+ 0.19	0.16	0.686
Secondary outcome measures								
Job satisfaction	6.62 (1.98)	6.43 (2.27)	− 0.09	6.80 (1.59)	6.40 (2.17)	− 0.21	0.20	0.660
Work fatigue	2.88 (1.14)	2.67 (1.29)	− 0.17	2.89 (1.34)	2.80 (1.32)	− 0.07	0.45	0.505
Work self-efficacy	3.97 (0.97)	4.12 (1.13)	+ 0.14	4.05 (1.10)	4.01 (1.20)	− 0.03	1.23	0.271
Days lost to illness	4.57 (9.91)	2.82 (5.66)	− 0.22	7.88 (15.64)	8.25 (14.45)	+ 0.02	0.26	0.615
Turnover intent	2.21 (1.10)	2.12 (1.14)	− 0.08	2.03 (1.04)	2.41 (1.26)	+ 0.33	**4.05**	**0.047**

^a^Group sample size reflects 13 cases lost to follow-up

^b^Group sample size reflects 8 cases lost to follow-up

^c^Includes covariates of age and years job tenure with employer

On the 6-month recall of days lost to illness there was a marked reduction in the intervention group (from 4.6 to 2.8) versus the control group (from 7.9 to 8.3), but this difference in means was not statistically significant due to the positive skew of these data (i.e., only a few workers reported a high number of days lost to ill health). However, a post-hoc analysis of the number of workers reporting more than 10 days of lost time (McNemar test, *p* < 0.05) showed a statistically significant worsening of sick days in the control group (those with > 10 days increased from 5 to 10 individuals) but not in the intervention group (those with > 10 days decreased from 4 individuals to 1 individual).

## Discussion

This study presents a novel employer-sponsored strategy to improve the working conditions for employees with chronic health problems using a group psycho-educational format. The conceptual framework for the intervention was based around concepts of self-efficacy and health self-management, but targeted to address challenges of communication, pacing, problem-solving, and job modification unique to the workplace setting. This registered randomized clinical trial (Clinicaltrials.gov: NCT01978392) provided a strong methodological framework to evaluate benefits of the MANAGE AT WORK intervention program. Though the project was ended prematurely due to an unexpected closing of the sponsoring research institute, preliminary results indicate an improvement in workplace engagement while other health and work outcomes are approaching statistically significant gains when compared with a wait-list control group.

This study adds to the growing literature on the struggles of aging or ill workers to stay on the job despite recurrent or episodic symptoms, but workplace intervention strategies for this population have been sparse in the occupational rehabilitation literature [44]. While much of the existing research has focused on quantifying health effects on work productivity, other research has been helpful to illustrate the more subjective struggles of workers to maintain social relations, negotiate regarding accommodations, develop a realistic understanding of personal capabilities, and to demonstrate an appropriate level of assertiveness and knowledge to overcome organizational barriers [46, 47]. Employers may find it difficult to provide a supportive work environment for workers with chronic health conditions when symptoms are intermittent and when there may be no explicit request for accommodation. As reported in previous studies of chronic medical conditions among working populations [48], many participants reported multiple comorbid health conditions, often involving multiple body parts and systems. The most prevalent problems related to widespread musculoskeletal pain.

One broad indication from the study is the relative success of recruiting at-risk employees, making meetings convenient and safe for participants, fostering discussion, and encouraging workplace self-management strategies without any reported incidents of negative interactions or adversarial relations at work. Thus, it may be possible to expand on workers’ efforts to obtain needed assistance and manage their workloads responsibly without disrupting employers and co-workers. One limitation of this approach is its reliance on workers to make or request adaptations in the workplace. The communication module of the training was built around the idea of promoting assertive communication about health, but this was balanced by the realities of working relationships, organizational culture, and workplace codes of conduct. Similarly, the job modification module was designed to make better use of existing leeway and flexibility using a problem-solving approach, while considering potential organizational or task-related barriers and constraints. Some workplace accommodation requests require more formal medico-legal processes, but the focus of the intervention was on informal types of job modification and assistance shown to be key for this working population with more episodic and transient problems [23].

The two primary outcome measures were work engagement and work limitations. Work engagement was the outcome measure that showed the greatest responsiveness to the intervention program. This construct was introduced in recent years to capture a more qualitative element of job satisfaction and is defined as “…a positive, fulfilling, work-related state of mind that is characterized by vigor, dedication, and absorption” [38]. For workers with chronic or episodic health problems, negative feelings about work may take the form of burn-out, exhaustion, feeling less capable and isolated, and experiencing a general disengagement from work that is well captured by this psychological construct. Our self-management intervention may address these beliefs by identifying more options for positive change, finding ways to communicate needs effectively to managers and co-workers, and building a sense of mastery to overcome future challenges. As such, the intervention may have led workers to feel more interested and active in their day-to-day work and less absorbed by growing health problems and feelings of helplessness.

The other primary outcome measure for the study was the Work Limitations Questionnaire (WLQ). In contrast to work engagement, the WLQ did not show improvement in response to the intervention in our preliminary analyses. This measure has been very instrumental in research and evaluation to quantify the work productivity losses attributable to various medical conditions [9, 15, 35], but it has only begun to be used as an individual-level metric for evaluating intervention effectiveness. The intervention failed to improve participants’ perceived ability to meet time pressures or perform physically demanding aspects of their work relative to the control group. One possible explanation is that the WLQ was not sufficiently responsive to short-term intervention, as the measure has shown statistically significant improvements in only one published trial (for depression) [49, 50]. Another possibility is that workers with chronic health concerns are less concerned about their raw productivity output than about their psychological well-being. The uptake of future workplace interventions for workers with chronic health conditions may depend on showing improved well-being while also enhancing productivity.

An important practical implication of the study is that employers can provide health coaching or other educational or counseling programs to workers with chronic or episodic illness in the same vein as other types of workplace health promotion (e.g., smoking cessation or weight loss programs). In this example, groups were facilitated by Employee Assistance Program (EAP) counselors, who were specially trained to deliver the intervention program. An advantage of this approach was that EAP counselors had both a strong mental health training background but also understood issues of organizational culture, workplace relationships, and disability employer regulations. A second implication of our results is that workers did experience some increased attachment to their jobs as a product of a facilitated group intervention addressing health-related challenges. Providing more options for dealing with workplace challenges and a greater sense of mastery may improve the dedication and commitment of workers with chronic health conditions.

Our study is not without limitations. The premature cessation of funding on the project led to a smaller sample size (119 vs. 300) and a reduced statistical power to show significant differences between groups. In terms of the intervention itself, an organizational component may have reinforced or strengthened the self-management and coping efforts of workers. Using cluster randomized designs may help to reduce the potential for cross-contamination between intervention and control groups in future studies. Most participants reported multiple chronic conditions, so the effect of overlapping comorbidities was difficult to assess. A shared effort that places equal responsibility on workers and supervisors to solve problems would place less onus on workers and more emphasis on organizational factors that can be changed.

Participating organizations may have been more apt than the average employer to allow workers to communicate with managers and peers about health concerns without any threat of retaliation or punitive treatment, though this is certainly an area of concern for more widespread implementation. The study sample included only a few occupational and industry groups, and effectiveness of the intervention may vary by occupation and industry. Future studies might combine this type of program with other organizational efforts (e.g., supervisor training, communications from management, ergonomic evaluations, peer coaches) to respond to the problem more completely. Another future direction would be to develop readiness assessment tools for employers to participate in such programs targeting workers with chronic health conditions.

In conclusion, this study demonstrates the initial feasibility of offering an employer-sponsored psycho-educational program to workers with chronic physical health conditions, and participation in this program resulted in improved levels of work engagement. Workers with chronic health conditions represent a fast-growing subset of the workforce in the US and elsewhere, and novel intervention strategies involving employer partnerships are sorely needed. We conclude that a group psycho-educational program is a feasible and effective way to improve worker engagement for those workers with chronic health conditions. Further efforts to develop and test employer-sponsored programs of this type should be pursued in different jurisdictions, contexts, and medical conditions to understand feasibility and effectiveness for more widespread dissemination.

## Data Availability

A deidentified dataset for research purposes is available from the authors.
